# Tropical Aquatic Archaea Show Environment-Specific Community Composition

**DOI:** 10.1371/journal.pone.0076321

**Published:** 2013-09-25

**Authors:** Cynthia B. Silveira, Alexander M. Cardoso, Felipe H. Coutinho, Joyce L. Lima, Leonardo H. Pinto, Rodolpho M. Albano, Maysa M. Clementino, Orlando B. Martins, Ricardo P. Vieira

**Affiliations:** 1 Instituto de Bioquímica Médica, Universidade Federal do Rio de Janeiro, UFRJ, Rio de Janeiro, Rio de Janeiro, Brazil; 2 Departamento de Genética, Universidade Federal do Rio de Janeiro, UFRJ, Rio de Janeiro, Rio de Janeiro, Brazil; 3 Centro Universitário Estadual da Zona Oeste - UEZO, Rio de Janeiro, Rio de Janeiro, Brazil; 4 Instituto Nacional de Metrologia, Qualidade e Tecnologia, INMETRO, Duque de Caxias, Rio de Janeiro, Brazil; 5 Departamento de Bioquímica, Universidade do Estado do Rio de Janeiro, UERJ, Rio de Janeiro, Rio de Janeiro, Brazil; 6 Instituto Nacional de Controle de Qualidade em Saúde, Fundação Oswaldo Cruz - FIOCRUZ, Rio de Janeiro, Rio de Janeiro, Brazil; Universidad Miguel Hernandez, Spain

## Abstract

The Archaea domain is ubiquitously distributed and extremely diverse, however, environmental factors that shape archaeal community structure are not well known. Aquatic environments, including the water column and sediments harbor many new uncultured archaeal species from which metabolic and ecological roles remain elusive. Some environments are especially neglected in terms of archaeal diversity, as is the case of pristine tropical areas. Here we investigate the archaeal composition in marine and freshwater systems from Ilha Grande, a South Atlantic tropical environment. All sampled habitats showed high archaeal diversity. No OTUs were shared between freshwater, marine and mangrove sediment samples, yet these environments are interconnected and geographically close, indicating environment-specific community structuring. Group II *Euryarchaeota* was the main clade in marine samples, while the new putative phylum Thaumarchaeota and LDS/RCV *Euryarchaeota* dominated freshwaters. Group III *Euryarchaeota* , a rare clade, was also retrieved in reasonable abundance in marine samples. The archaeal community from mangrove sediments was composed mainly by members of mesophilic Crenarchaeota and by a distinct clade forming a sister-group to Crenarchaeota and Thaumarchaeota. Our results show strong environment-specific community structuring in tropical aquatic Archaea, as previously seen for Bacteria.

## Introduction

It is well established that Archaea are widely distributed and numerically significant in aquatic ecosystems [1-4]. Microbial biogeographic patterns, however, are still a mystery for many taxa. Since molecular methods started to be applied to the study of uncultivated microbial communities [5], knowledge of their ecology in aquatic systems has been significantly increased [6-9] but archaeal diversity and distribution remain poorly known.

Marine environments are the most thoroughly studied among aquatic ecosystems concerning archaeal diversity. Group II *Euryarchaeota* are common in euphotic zones of open ocean waters and in shallow coastal zones [10-12] while rare taxa like Groups III and IV *Euryarchaeota* [13,14] can occur in deep ocean waters. There are no cultivated representatives of Groups II, III and IV *Euryarchaeota* , and their specific metabolism remains elusive [15]. Group I Archaea comprises abundant organisms in the mesopelagic zone and their substantial contribution to global nitrogen and carbon cycles has been demonstrated [16]. Group I Archaea was initially classified as mesophilic Crenarchaeota [1,2], albeit they emerged only as a sister group of hyperthermophilic Crenarchaeota in phylogenetic trees. Isolation and complete genome sequencing of members of this group, as *Nitrosopumilus* *maritimus* from marine aquarium sediment [17,18] and *Cenarchaeum* *symbiosum* from a marine sponge [19,20], shed light on its ecology and deep phylogenetic study of this group strongly suggests that Group I Archaea form an exclusive division within the Archaea domain, the Thaumarchaeota [21-23].

Less documented than marine habitats, freshwater environments have been shown to host new highly diverse archaeal taxa [24,25]. Its remarkable richness [26-28] has been suggested to be due to a great number of niches provided by micro-habitats associated with particulate material [29]. One of the most abundant archaeal lineages found in freshwater are the LDS/RCV *Euryarchaeota* , initially described in Lake Dagow sediment (LDS) and in rice wetlands (RCV) [24,30]. However, as seen with Groups II, III and IV, there are no cultivated representatives and their ecological roles are yet to be understood. Microbial community from freshwater samples from Amazon River, Brazil and Lake Gatun (Panama) were analyzed by metagenomic approach, and show the presence of Thaumarchaeota [31,32]. Though less abundant in whole Amazon River prokaryotic community, these sequences are mainly recruited by *Nitrosopumilus* *maritimus* genome, an ammonium-oxidizing archaeon, indicating an important role of this archaeal group in biogeochemical cycling in freshwater environments [4,32,33].

Most sediment Archaea and Bacteria seem to be autochthonous and not merely accumulated from the pelagic zone [34]. Theoretically, like in the water column, sediment communities should contain many ubiquitous, broadly distributed prokaryotic groups since environmental conditions (temperature, nutrient availability and supply, and pressure) are considered to be generally similar over wide tracts of the seabed [35]. In fact, salinity and oxic-anoxic conditions seem to be the key environmental factors structuring archaeal communities in both water and sediments [4], despite geographical location.

Emerging environmental sequences coming from poorly studied environments have been changing archaeal tree and the knowledge about its distribution [36]. Considering that, the study of tropical aquatic environments, of which there is scarce knowledge, can add significant contributions to the understanding archaeal biology [37-39]. The tropical South Atlantic Ocean and coastal zones are still poorly studied environments regarding microbial diversity and distribution [40,41]. They are also strongly influenced by the Atlantic rain forest, a hotspot of biological diversity, which contributes with a substantial amount of organic and inorganic material to marine ecosystems [42-44]. Although the response of bacterial communities to salinity changes in river to coast gradients has been accessed [45,46], archaeal behavior in these conditions remain elusive. The aim of this study was, therefore, investigate for the first time archaeal composition in the tropical island Ilha Grande in Brazil, a protected area subjected to a very low anthropogenic impact in Brazil. The differences in community composition found here help to elucidate archaeal distribution in coastal tropical habitats.

## Materials and Methods

### Sampling

Water samples (5.8 Liters) were collected at 1m depth (except for the water spring, where superficial water was collected) and mangrove sediment samples (50g) were collected in a 50 mL falcon tube, on September 7, 2007, for DNA extraction. Samples were kept on ice until processed in the laboratory. The samples were collected in accordance with the Brazilian law (IN 154/2007 IBAMA, Brazilian Institute of Environment and Renewable Natural Resources) and we confirm that the field studies did not involve endangered or protected species.

**Figure 1:**
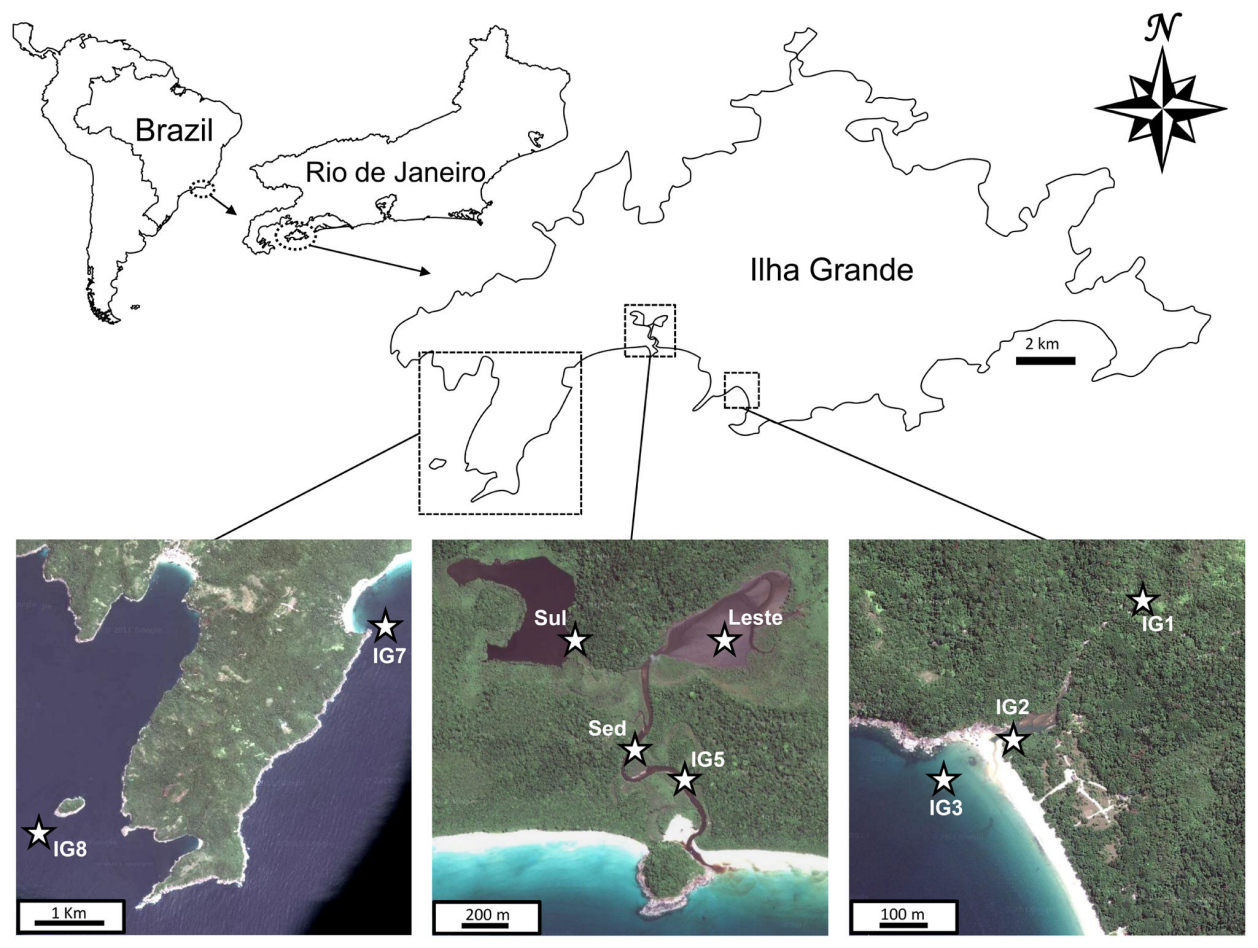
Map of the studied site and the nine sampled locations. IG1 – Parnaioca water spring; IG2 – Parnaioca river; IG5 – mangrove; IG3 – Parnaioca beach; IG7 – Aventureiros beach; IG8 – Meros; SedIG – sediment from mangrove channel; SedL – Leste lagoon entrance; SedS – Sul lagoon entrance.

The nine analysed sites, three freshwater, three marine and three mangrove sediment are shown in Figure 1: IG1- a water spring (23°10’57.00″ S/ 44°14’55.19″ W); IG2 Parnaioca river (23°11’21.33″ S/ 44°15’11.08″ W); IG3- Parnaioca beach (23°11’24.77″ S/ 44°15’15.07″ W), just where Parnaioca river flows into the sea; IG5- mangrove channel (23°10’26.98″ S/ 44°17’08.49″ W); IG7- Aventureiros beach (23°11’24.53″ S/ 44°18’58.06″ W); IG8- near Meros island (23°12’53.67″ S/ 44°21’55.03″ W). Sed – (23°10'25.14″S/ 44°17'16.14″W) superficial sediments of the mangrove channel; Leste – (23°10'6.34″S/ 44°17'0.97"W) 20 cm deep sediment at the center of Leste lagoon; Sul – (23°10'9.65″S/ 44°17'24.14″W) 20 cm deep sediment at the entrance of Sul lagoon.

### DNA extraction

The water samples were filtered through 0.22 µm SV Sterivex filters (Millipore, Bedford, MA, USA) after filtration through a 3.0 µm ester cellulose filter (Millipore, Bedford, MA, USA) to separate free-living microbes from larger organisms and particles. Total cellular nucleic acids were isolated from the free-living fraction by cell lysis with proteinase K and SDS, followed by phenol-chloroform extraction [47]. DNA integrity was checked on a 1% (w/v) agarose gel. DNA from sediment samples was extracted by an adapted protocol [48]. Briefly, 100 mg of sediment was washed with PBS and submitted to three cycles of freeze/thawing: -70°C for 2 min and 65°C for 2 min. Then, samples were incubated with 2% SDS for 10 min at 60°C. Subsequently, glass beads (0.1 mm) were added and the mixture was agitated for 80 sec at maximum speed in Bead Beater equipment (Mini-Bead-Beater; Bartlesville, Okla, USA) three times. Samples were centrifuged and the supernatant was recovered and subjected to phenol-chloroform extraction.

### 16S rRNA gene library construction

PCR was performed in 50 µl reaction mixtures (2.5 mM MgCl_2_, 0.2 mM deoxynucleoside triphosphates, 1 ng of each primer. µl^-1^, 2.5 U of high fidelity *Taq* DNA polymerase (Promega), 1× PCR buffer and 200 ng of each environmental DNA sample, using the universal archaeal primers 21AF (5'-TTCCGGTTGATCCTGCCGGA-3') [2] and 907RAB (5'-TTTGAGTTT MCTTAACTGCC-3') [49]. PCR amplification began with a 5 min denaturing step at 94°C which was followed by 30 cycles of 94°C for 30 seconds, 50°C for 90 seconds, and 72°C for 90 seconds. The final cycle was an extension at 72°C for 5 min. PCR products were concentrated and purified with a GFx PCR DNA and Gel Band Purification Kit (GE Healthcare) after electrophoresis on a 1% (w/v) agarose gel. Amplicons were cloned into the pGEM-T cloning vector (Promega) and used to transform electro-competent *E. coli* DH10B cells. Positive colonies were picked and frozen at -70°C. Nine archaeal 16S rRNA gene libraries were constructed from the different environmental DNA samples.

### Sequence analyses and taxa identification

For evaluation of the main archaeal groups in the samples, approximately 96 clones from each clone library were submitted to sequence analysis. Plasmidial DNA from each clone (400 ng) was prepared and PCR-sequencing reactions with primer 21AF were carried out using the DYEnamic ET terminator cycle-sequencing kit (GE Healthcare). Partial 16S rRNA sequences were obtained by capillary electrophoresis on a MegaBace1000 DNA analysis system (GE Healthcare). Electropherograms were transformed into Fasta format with Phred software [50] and sequences with less than 300 bp and chimeras were removed prior to further analysis using MOTHUR [51]. A total of 496 valid sequences with Phred score ≥ 20 were compared with sequences in the Ribosomal Database Project II [52]. Sequences were also analyzed by BLAST [53] searches in the GenBank database and were aligned with representative archaeal sequences obtained from public databases using ClustalW software [54]. The partial 16S rRNA gene sequences generated in this study have been deposited in GenBank under accession numbers JF835116-JF835611.

### Biodiversity and phylogenetic analyses

Sequences were clustered as OTUs at an overlap identity cutoff of 97% and 80% by MOTHUR. Richness and diversity statistics including the nonparametric richness estimator Chao1 and the Shannon diversity indexes were calculated. OTUs diversity and community overlap were also examined using rarefaction analysis and Venn diagrams. Phylogenetic trees were constructed with reference sequences by the Maximum Likelihood algorithm based on distances calculated by the Kimura-2 method. This analysis was performed with the MEGA5 program [55] and bootstrap analysis with 1000 replications was used. Tree topology and distribution of hits along the tree (without reference sequences) were uploaded to the UniFrac computational platform [56]. UniFrac is a beta diversity metric analysis that quantifies community similarity based on phylogenetic relatedness. In order to visualize distribution patterns of bacterial communities we used the UniFrac metric to perform PCoA highlighted by significance.

### Statistical comparison between archaeal 16S rRNA libraries

We used ∫-LIBSHUFF statistical method to determine differences in library composition in the communities from which they are derived [57]. This method uses Monte Carlo methods to generate homologous and heterologous coverage curves. Sequences were randomly shuffled 10,000 times between samples prior to the distance between the curves being calculated using the Cramér-von Mise statistic test. The DNADIST program of the PHYLIP package, with the Jukes-Cantor model for nucleotide substitution, was used to generate the distance matrix analyzed by ∫-LIBSHUFF.

## Results and Discussion

### Clone library comparison

Small subunit ribosomal RNA libraries were sequenced form freshwater, marine water and brackish sediments. Although libraries were far from saturation when analyzed at 97% sequence identity, and the actual archaeal diversity in these samples may not have been fully characterized, the number of clones sequenced allows the detection of main archaeal groups in the sample. Total number of sequences, OTUs, richness (Chao 1) and diversity (H’) indexes calculated by MOTHUR software from each site are shown in Table S1. We also grouped freshwater (IG1, IG2, IG5), marine (IG3, IG7, IG8) and sediment (Sed, Leste, Sul) libraries to perform these calculations. There were no significant differences in archaeal richness and diversity when comparing the three habitats: freshwater, seawater and sediments. However, the analysis of each sampled site shows that Parnaioca river, Parnaioca beach and Sul lagoon sediment had remarkable higher richness and diversity.

**Figure 2:**
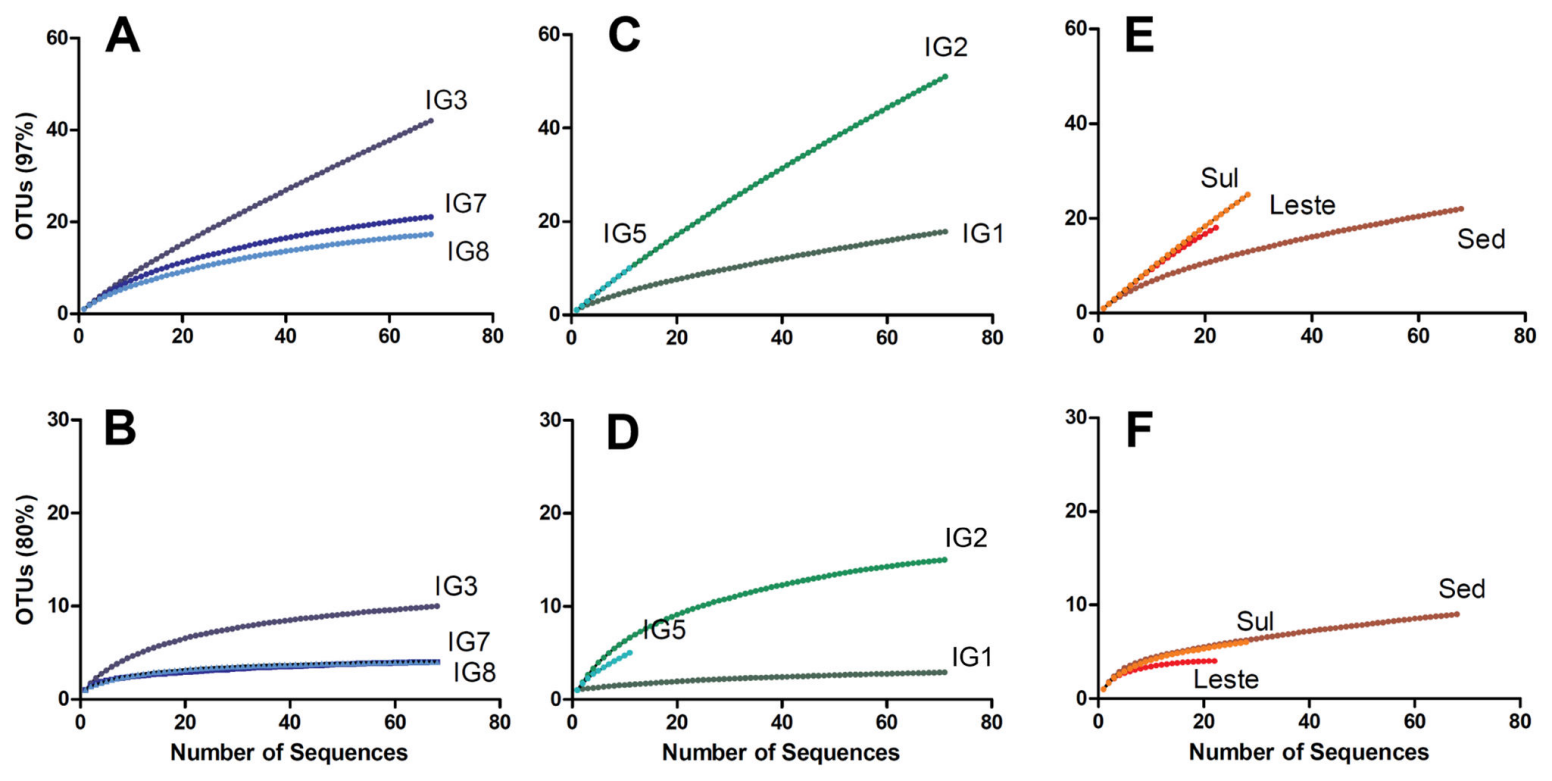
Rarefaction analyses of 16S rDNA clone libraries at 97% (A, C and E) and 80% (B, D and F). In A and B each marine water library is plotted, in C and D, each freshwater and in E and F each sediment.

Rarefaction curves at a high phylogeny resolution (97%) confirm that archaeal communities from Parnaioca river, Parnaioca beach and mangrove sediment from Sul lagoon are more diverse than the other sites (Figure 2A, C and E). At 80% resolution, all libraries tended to a plateau (Figure 2B, D and F), showing that representatives of all archaeal phyla, but not all species, were sampled, as expected by the applied methodology and sequencing effort.

**Figure 3:**
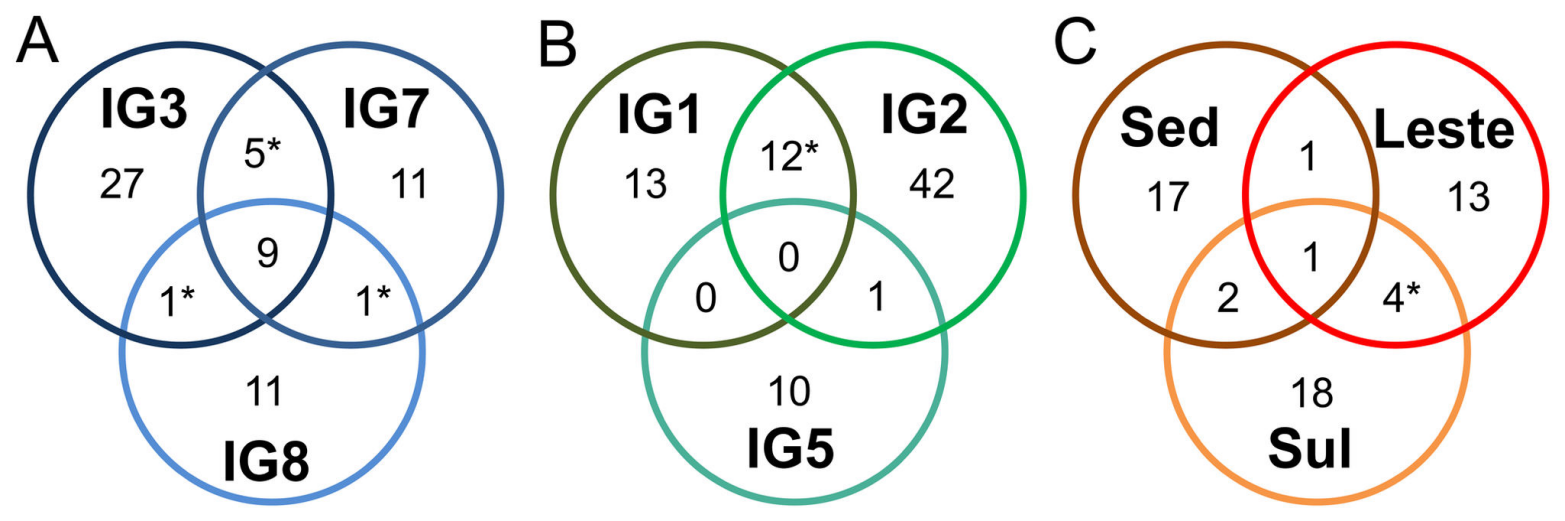
Venn diagram at 97% identity. A: Seawater, B: Freshwater, C: Sediment. IG1 – Parnaioca water spring; IG2 – Parnaioca river; IG5 – mangrove; IG3 – Parnaioca beach; IG7 – Aventureiros beach; IG8 – Meros; SedIG –mangrove channel; Leste – Leste lagoon center; Sul - Sul lagoon entrance. Asterisks shown for ∫-LIBSHUFF comparisons with p value >0.0001.

Venn diagrams showed that no OTUs are shared between freshwater, seawater and sediments at the species level (97%). However, within each different ecosystem (freshwater, marine and sediments) many OTUs are shared (Figure 3A, B and C). Spatial heterogeneity among sites of the same kind of environment was lower than what was observed for bacteria, with a greater number of archaeal than bacterial OTUs shared within fresh or seawater samples [46]. The ∫-LIBSHUFF analysis shows that samples sharing a great number of OTUs in Venn diagrams (labeled with an asterisk) are also considered as similar in composition (p values>0.0001). That is the case of Parnaioca river and water spring samples, mangrove sediment samples from Sul and Leste lagoons and among the three seawater samples.

**Figure 4:**
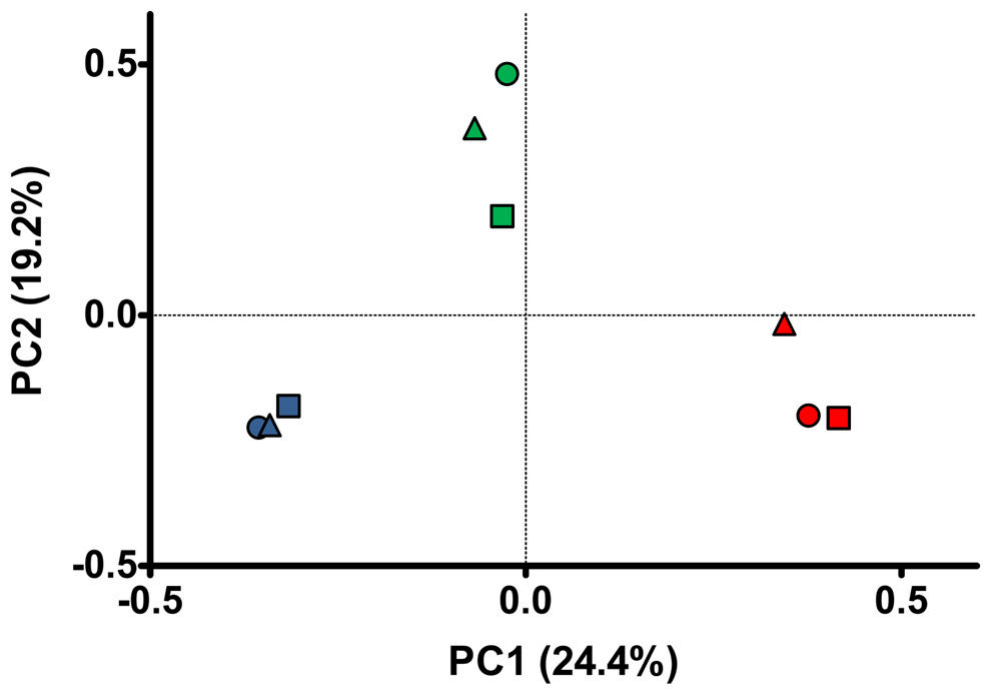
Match between archaeal communities in freshwater, seawater and sediment libraries. Principal coordinates plots (PCoA) were generated using the pair wise unweighted UniFrac distances. Freshwater samples in circles, marine samples in squares, and sediments in triangles. Parnaioca Spring (green triangle); Parnaioca river (green circle); Mangrove water (green square); Parnaioca beach (blue triangle); Aventureiros beach (blue circle); Meros Island (blue square); SedIg (red triangle); SedLeste (red circle); SedSul (red square).

UniFrac is a beta diversity metric that quantifies community similarities based on phylogenetic relatedness [58]. In the scatter plot of the first two principal coordinates of the UniFrac analysis (Figure 4), PC1 and PC2 explained 24.4% and 19.2% of the data variation, respectively. Marine libraries were separated from freshwater ones in the plot by PC2. The three marine libraries grouped together showing a high similarity with each other, whereas freshwater samples were dispersed in the plot and seem to be more different among them. Additionally, the mangrove water library clustered between freshwater and marine samples along the PC2 axis that divides saline from the other freshwater environments. Sediment libraries showed some heterogeneity among them and were dispersed in the same way as marine libraries along the PC2 axis, even though these sediments were covered by freshwater. They were, however, separated by the PC1 axis showing they substantially diverge from planktonic samples. UniFrac results corroborate the ∫-LIBSHUFF analysis, wherein marine libraries and the two lagoons reached high p values.

### Phylogenetic Analysis

**Figure 5:**
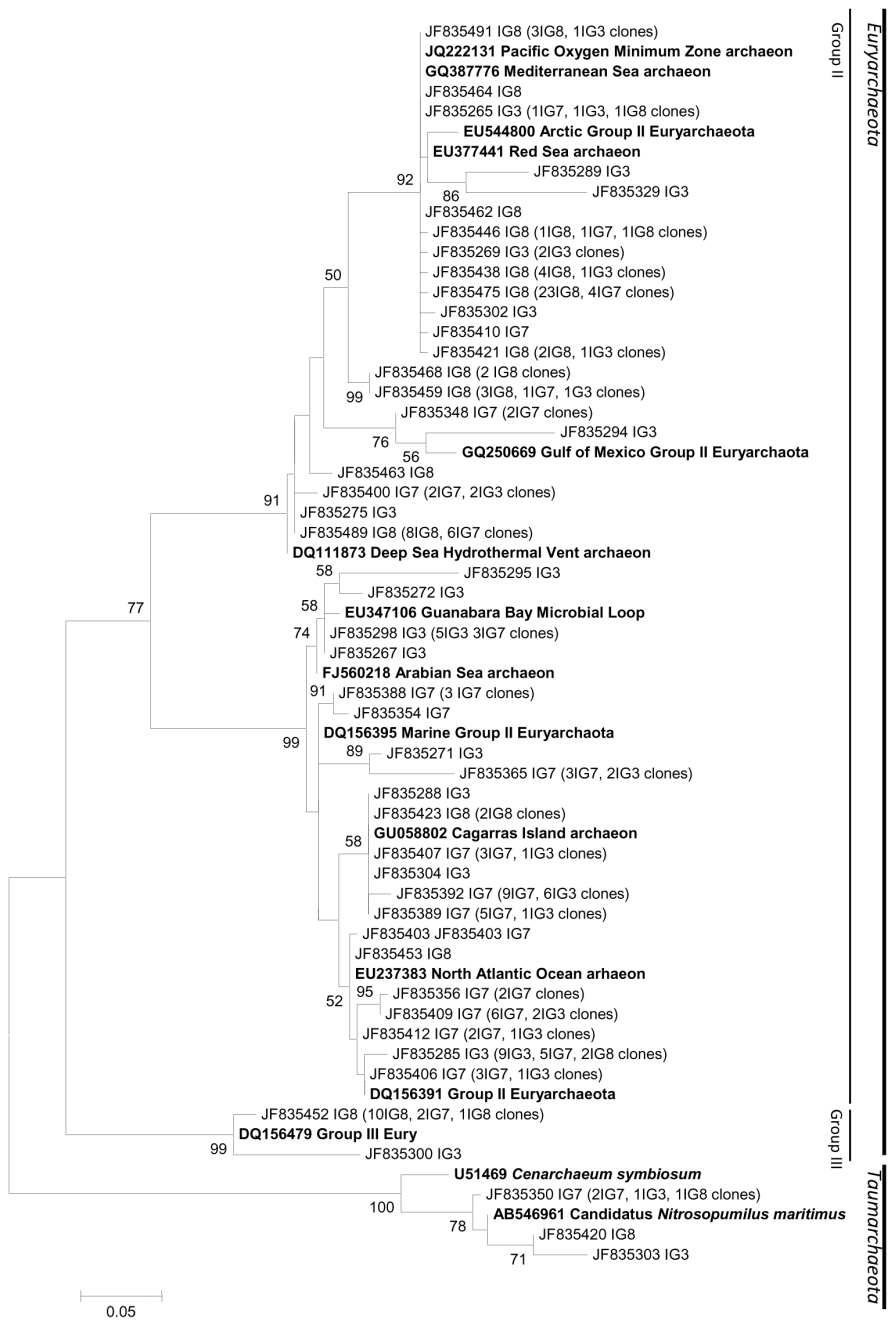
Marine Phylogenetic archaeal tree. Reference sequences from GenBank (**in bold**). OTUs were defined by using a distance level of 3% by using the furthest neighbor algorithm in MOTHUR. The tree topology is based on maximum likelihood and bootstrap analysis was performed with 1000 replications. Bootstrap value <50 are not shown.

**Figure 6:**
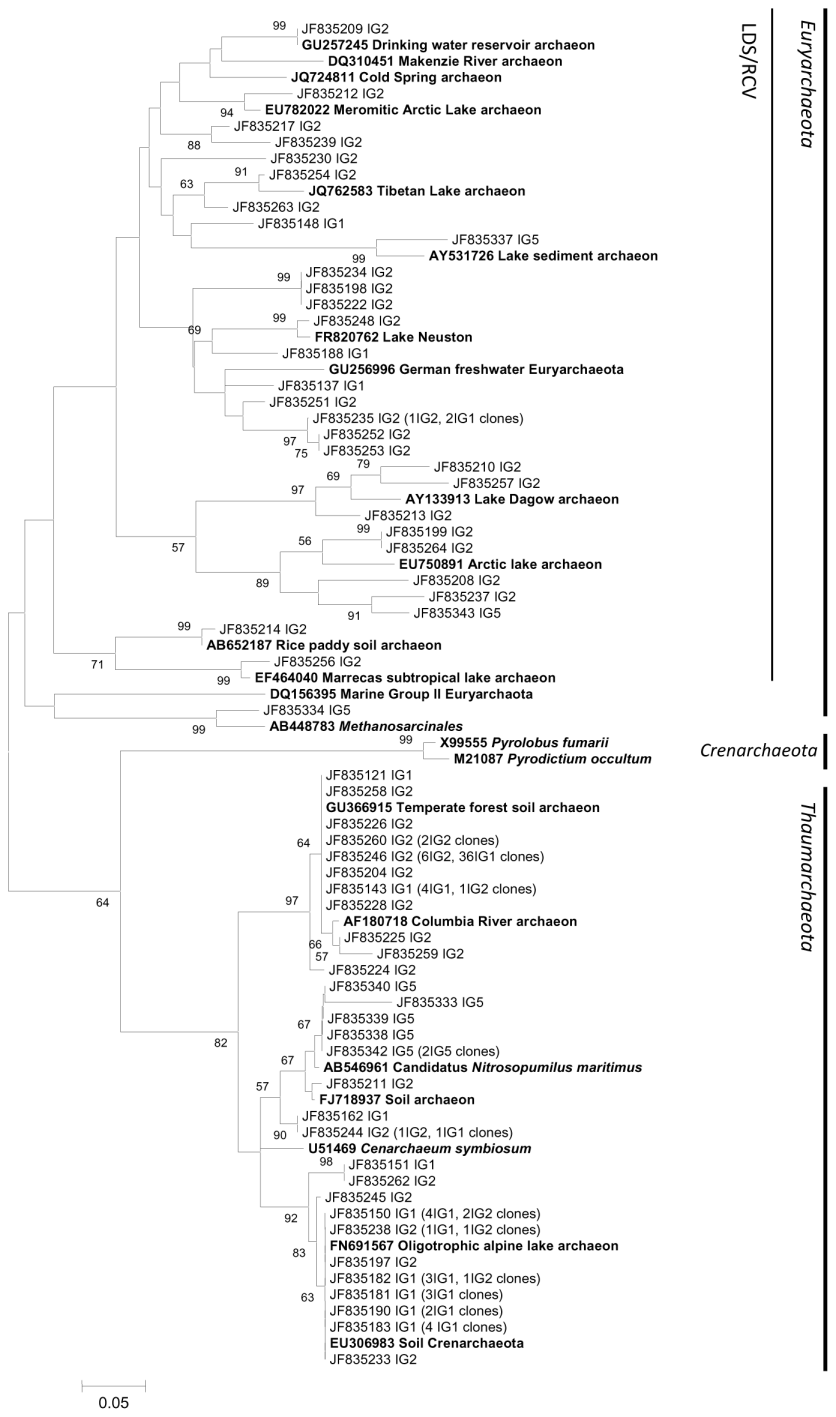
Freshwater Phylogenetic archaeal tree. Reference sequences from GenBank (**in bold**). OTUs were defined by using a distance level of 3% by using the furthest neighbor algorithm in MOTHUR. The tree topology is based on maximum likelihood and bootstrap analysis was performed with 1000 replications. Bootstrap value <50 are not shown.

**Figure 7:**
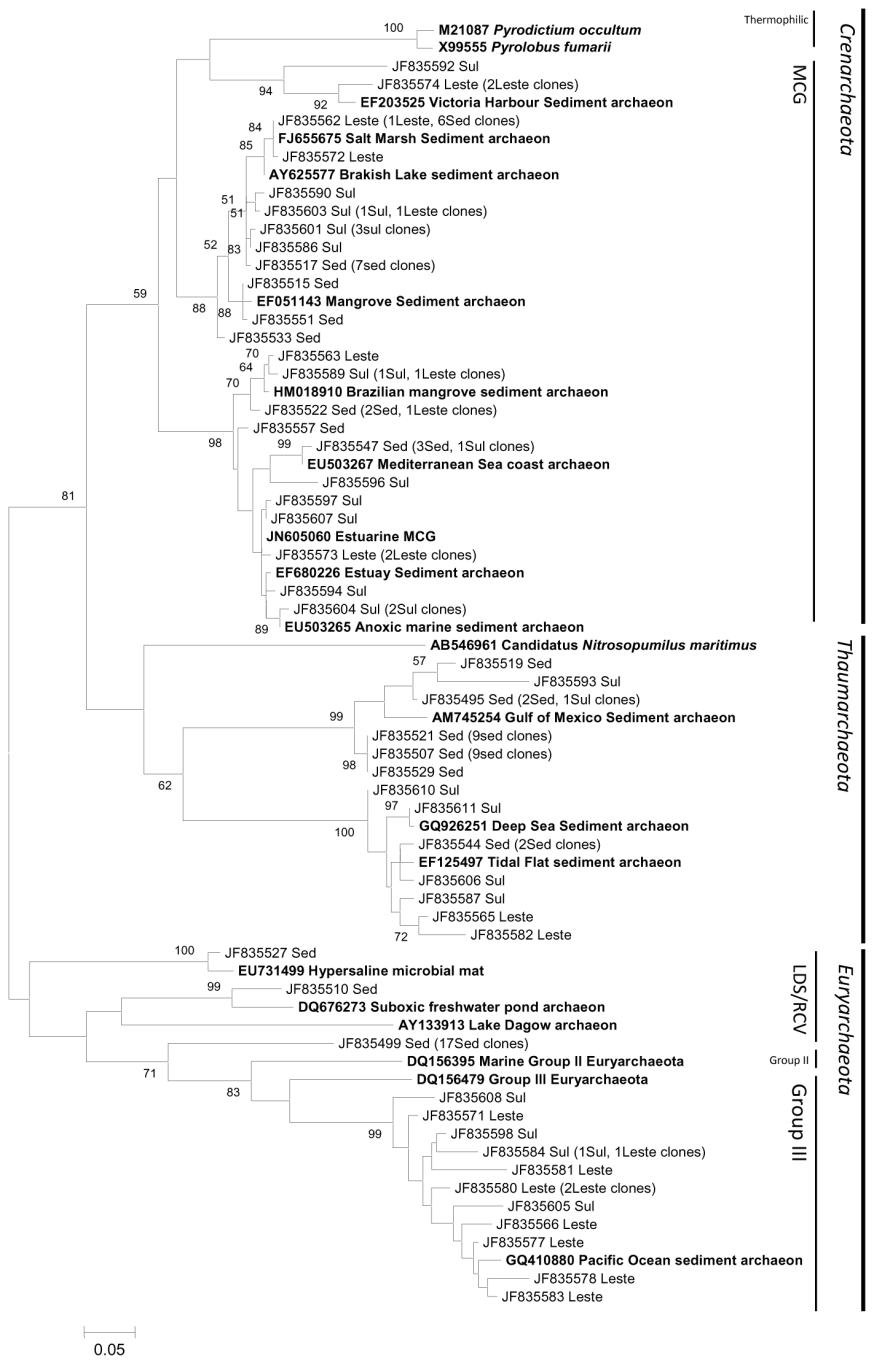
Sediment Phylogenetic archaeal tree. Reference sequences from GenBank (**in bold**). OTUs were defined by using a distance level of 3% by using the furthest neighbor algorithm in MOTHUR. The tree topology is based on maximum likelihood and bootstrap analysis was performed with 1000 replications. Bootstrap value <50 are not shown.

Since there are no shared OTUs between seawater, freshwater, and sediments, we performed three independent phylogenetic tree constructions (Figures 5, 6 and 7, respectively). The marine tree (Figure 5) shows that the main archaeal clade in Ilha Grande seawater survey is composed by Group II *Euryarchaeota* that has been shown previously to be abundant in marine coastal and superficial waters [10,15]. These *Euryarchaeota* were affiliated to other environmentally widespread sequences retrieved from the North Atlantic Ocean, Red Sea, Gulf of Mexico, Cagarras Island in Brazil and the Arctic Sea, showing that these are ubiquitous marine organisms. We also found two OTUs, one with 14 clones, closely related to members of Group III *Euryarchaeota* , a rare group in marine waters which is usually only detected in deep sequencing studies [14]. This group showed affiliated OTUs in Parnaioca and Aventureiros beaches and especially in Meros Island, where 11 clones were retrieved, a surprising observation since this group is always rare and found in deep ocean samples [14,59]. Clones related to this group were also described more commonly in anoxic sediments, lake waters and associated to corals [24,30,60,61]. Three OTUs within marine samples belong to the Thaumarchaeota phylum, being closely related to *C* *. symbiosum* and to *N* *. maritimus* . Notably, there is no sample-specific group in any archaeal phylum, showing the homogeneity among the marine samples, as was also indicated by Venn diagram and ∫-LIBSHUFF analyses.

In the freshwater sample, almost half of the clones belong to the Thaumarchaeota phylum (Figure 6), forming a unique cluster comprising sequences also retrieved from soil and freshwater lakes. In spite of the low salinity, these phylotypes are closely related to marine ammonia-oxidizing *Thaumarchaeota* *N* *. maritimus* , and not to freshwater and brackish members of this clade like *Nitrosoarchaeumliminia* [62]. The high representation of *Thaumarchaeota* sequences in this study corroborates the hypothesis of great importance of this clade to nitrogen cycling in freshwater environments [32,33]. Compared to marine groups, *Thaumarchaeota* from freshwater show lower *AmoA* gene phylogenetic diversity, in contrast to the high diversity found in 16S gene studies, what could be due to biased sampling effort [63]. There are no OTUs belonging to Groups II and III *Euryarchaeota* in freshwater libraries but only a single sequence related to *Methanosarcinales*. A great group of typical freshwater *Euryarchaeota* , the LDS/ RCV cluster, was also detected. Even though most OTUs within this group are from Parnaioca river, the remaining OTUs are also found in the other two freshwater samples. The lack of specific clusters of Parnaioca river in the freshwater tree together with its high diversity could suggest that part of the riverine archaeal community could be of soil origin [29]. High diversity in freshwater systems, for archaea and other taxa, has been previously described, but the reasons that lead to this are still debated [4,64].

Mangrove sediments showed the most complex archaeal community at the phylum or sub-phylum level, composed by Thaumarchaeota, Crenarchaeota, Group III and LDS/RCV *Euryarchaeota* (Figure 7). Members of Crenarchaeota form a large clade distant related to hyperthermophilic species, such as *Pyrolobus fumarii* and *Pyrodictium occultum*. Within this group, phylotypes from the three sediment libraries are closely related to the ubiquitous Miscelaneous Crenarchaota Group (MCG), retrieved worldwide from estuarine, coastal, mangrove and lake sediments. This group is remarkably high in anoxic, low energy environments, but seem not be enrolled in sulphate reduction and methane oxidation [65]. The close phylogenetic relationship between OTUs from this clade and clones retrieved from high salinity sediments could indicate that the oxic state of the sediments, instead of salinity and temperature, is the most important feature structuring archaeal communities in sediments [66]. A large clade distant related to *N* *. maritimus* was identified, possibly comprising a new sediment *Thaumarchaeota* clade. This group could be enrolled to ammonia oxidation in first layers of sediment, as archaeal anaerobic ammonium oxidation was not reported to date [67]. Group III *Euryarchaeota* representatives were found in sediments from Sul and Leste lagoons, while members of LDS/RCV cluster were found only in Sed library. The absence of methanogens in Ilha Grande mangrove sediment libraries can be due to pH or low concentrations of electron acceptor like nitrate, ferric iron and sulfate [67].

The fact that the environment is a major determinant of the evolutionary relationships between members of the Bacteria and Archaea domains suggests that each habitat is home to some extremely well-adapted specialized lineages, a hypothesis that comes from indicator-species used in macroorganism ecology [68,69]. This hypothesis is supported by the study of Auguet et al., 2010 [4], which found at least one indicator lineage for each habitat studied. Ilha Grande bacterial and archaeal communities seem to follow this paradigm, with many habitat-specific clusters [46]. Comparative studies concerning the distribution, community structure patterns and environmental factors modulating uncultured populations are essential to understand archaeal biology.

## Supporting Information

Table S1 **Archaeal richness and diversity.**
(DOC)
