# Supplementary material for: Tropical Aquatic Archaea Show Environment-Specific Community Composition
Source: PLoS One. 2013 Sep 25;8(9):e76321. doi: 10.1371/journal.pone.0076321 (PMC3783403; doi:10.1371/journal.pone.0076321)
Supplement: Table S1 — Archaeal richness and diversity. (DOC) [file pone.0076321.s001.doc]

Table S1**.**  Archaeal richness and diversity.

^a^SG ^b^OTUs Chao1 ^c^H’

FW 160 78 255 3.8

IG1 78 19 45 1.8

IG2 71 51 158 3.6

IG5 11 10 28 2.2

SW 218 65 237 3.4

IG3 68 42 161 3.3

IG7 75 22 31 2.6

IG8 75 18 23 2.2

SD 118 56 119 3.5

Sed 68 22 41 2.5

Sul 28 25 151 3.1

Leste 22 18 36 2.8

^a^ Number of sequences deposited at GenBank (SG).

^b^ Number of unique OTUs (Operational Taxonomic Unit) defined by using the furthest neighbor algorithm in MOTHUR at 97% similarity.

^c^ Shannon-weaver index of diversity (H’).
